# Productos comunicacionales para la prevención de la COVID-19 promovidos por los gobiernos de América Latina y el Caribe

**DOI:** 10.26633/RPSP.2021.111

**Published:** 2021-11-19

**Authors:** Daniela Moyano, Lina Lay Mendivil

**Affiliations:** 1 Universidad Nacional de La Matanza Universidad Nacional de Córdoba Buenos Aires Córdoba Argentina Universidad Nacional de La Matanza, Buenos Aires, Argentina. Universidad Nacional de Córdoba, Córdoba, Argentina.; 2 Grupo de Investigación en Nutrición y Sistemas Alimentarios Saludables y Sostenibles INSAS-UTP Ciudad de Panamá Panamá Grupo de Investigación en Nutrición y Sistemas Alimentarios Saludables y Sostenibles INSAS-UTP, Ciudad de Panamá, Panamá.

**Keywords:** Comunicación en salud, infecciones por coronavirus, programas de gobierno, América Latina, región del Caribe, Health communication, coronavirus infections, government programs, Latin America, Caribbean region, Comunicação em saúde, infecções por coronavirus, programas governamentais, América Latina, região do Caribe

## Abstract

**Objetivo.:**

Caracterizar el contenido de los productos comunicacionales dirigidos a la población general para la prevención de la COVID-19 promovidos por los gobiernos de América Latina y el Caribe (ALC).

**Métodos.:**

Estudio descriptivo, trasversal y exploratorio de los productos comunicacionales publicados hasta el 28 de febrero del 2021 en los sitios web oficiales de los ministerios de salud de los 47 países y territorios de ALC para la prevención de la COVID-19. Se emplearon métodos cuantitativos y cualitativos a partir de dimensiones recomendadas por la Organización Mundial de la Salud (OMS) y un enfoque de determinantes sociales de la salud (DSS). Se utilizó la técnica de análisis de contenido y triangulación por observador.

**Resultados.:**

Se analizaron 3 253 productos informativos; en los 47 países y territorios de ALC, al menos un producto incluyó alguna recomendación de la OMS y en 46 se abordó alguna de las categorías y subcategorías relacionadas con los DSS intermedios. De los DSS estructurales, se aplicó el enfoque de género y alguna lengua indígena en al menos un producto de solo 4 y 7 países, respectivamente. En 35 países se encontró al menos un producto con información errónea y en 13 no se abordó la infodemia.

**Conclusiones.:**

Se encontró una cantidad considerable de productos comunicacionales para la prevención de la COVID-19, pero no reflejaban un adecuado enfoque de diversidad y salud integral, y mostraban vacíos y errores de comunicación. Los países deben revisar sus políticas de comunicación en salud en el contexto de la pandemia.

## Abstract (en)

**Objective.:**

Characterize the content of communication products for COVID-19 prevention promoted by governments in Latin America and the Caribbean (LAC) for the general public.

**Methods.:**

A descriptive, cross-cutting, and exploratory study of the communication products for COVID-19 prevention published up to 28 February 2021 on official websites of the ministries of health of the 47 countries and territories of LAC. Quantitative and qualitative methods based on the dimensions recommended by the World Health Organization (WHO) were employed, together with a social determinants of health (SDH) approach. The content analysis technique and investigator triangulation were used to improve internal validity.

**Results.:**

3 253 information products were analyzed. In 47 countries and territories in LAC at least one product included a WHO recommendation; and in 46 at least one category or subcategory related to intermediate SDHs was addressed. Regarding structural SDHs, a gender approach was used in at least one product in only four countries, and an indigenous language in only seven countries. In 35 countries, at least one product was found to have erroneous information and in 13 countries the infodemic was not addressed.

**Conclusions.:**

A substantial number of communication products for COVID-19 prevention were found, but they lacked an adequate diversity approach and comprehensive health approach, and they showed gaps and communication errors. The countries should review their health communication policies in the context of the pandemic.

## RESUMO

**Objetivo.:**

Caracterizar o conteúdo dos produtos de comunicação dirigidos à população em geral, para a prevenção da COVID-19, promovidos pelos governos da América Latina e do Caribe (ALC).

**Métodos.:**

Estudo descritivo, transversal e exploratório dos produtos de comunicação publicados até 28 de fevereiro de 2021 nos sites oficiais dos ministérios da Saúde de 47 países e territórios da ALC para a prevenção da COVID-19. Foram utilizados métodos quantitativos e qualitativos com base nas dimensões recomendadas pela Organização Mundial da Saúde (OMS) e enfoque nos determinantes sociais da saúde (DSS). Foram utilizadas as técnicas de análise de conteúdo e triangulação de investigador.

**Resultados.:**

Foram analisados 3 253 produtos de informação. Nos 47 países e territórios da ALC, pelo menos um produto incluiu alguma recomendação da OMS, e em 46, foram abordadas algumas das categorias e subcategorias relacionadas aos DSS intermediários. Dos DSS estruturais, foram utilizados enfoque de gênero e alguma língua indígena em pelo menos um produto de apenas 4 e 7 países, respectivamente. Em 35 países foi encontrado pelo menos um produto com informações errôneas, e em 13 não foi abordada a infodemia.

**Conclusões.:**

Foi encontrado um número considerável de produtos de comunicação para a prevenção da COVID-19; entretanto, eles não refletiam uma abordagem adequada à diversidade e à saúde integral, e apresentavam lacunas e erros de comunicação. Os países devem revisar suas políticas de comunicação em saúde no contexto da pandemia.

Desde la notificación del primer caso de COVID-19 en América Latina y el Caribe (ALC), esta enfermedad se ha propagado a todos los países y territorios del área (1) en un contexto en el que la desigualdad en el acceso a los servicios de salud sigue siendo un problema estructural (2).

Para disminuir las desigualdades es fundamental que la información relacionada con la salud sea adecuada, suficiente, clara, oportuna y accesible (3). Especialmente durante las crisis sanitarias, la comunicación en temas de salud influye en la toma de decisiones de las personas y, si los materiales comunicacionales son adecuados, pueden propiciar los cambios de comportamiento necesarios (4).

La pandemia de COVID-19 ha generado una explosión global de información (5), para lo que se ha acuñado el término infodemia: exceso abrumador de información, con una considerable presencia de datos inexactos, falsos o engañosos (6). En este contexto es imprescindible profundizar en el contenido de las campañas de comunicación para ayudar a contener la propagación de informaciones sin fundamento científico y contribuir al manejo de la pandemia desde una correcta comunicación de riesgo (7, 8), algo estrechamente relacionado con la alfabetización en salud (9).

Investigaciones previas sobre la comunicación a la población durante la pandemia indican que varios medios y portales informativos compartieron información falsa (10) o inexacta (11), cuyo impacto negativo resulta mayor en grupos vulnerables, que luego sufren graves consecuencias a causa de esa mala información (12). Por otro lado, existe la preocupación de que los mensajes emitidos durante la pandemia no estén llegando a una parte importante de la población en riesgo (13). Los materiales comunicacionales y los mensajes deben estar diseñados de modo que nadie quede excluido (14).

Si bien existen antecedentes científicos sobre comunicación de riesgo durante los brotes de ébola, zika y fiebre amarilla (15), faltan investigaciones concretas sobre el contenido de las campañas de comunicación generadas desde los gobiernos de ALC durante la actual pandemia de COVID-19.

En este trabajo se hace una caracterización del contenido de los productos comunicacionales dirigidos a la población general para la prevención de la COVID-19 promovidos por los gobiernos de ALC.

## MATERIALES Y MÉTODOS

Se realizó un estudio descriptivo, trasversal y exploratorio de los productos comunicacionales publicados en sitios web oficiales de los gobiernos de ALC (16), con un enfoque centrado en la prevención, según las recomendaciones específicas para el público general (17), la comunicación de riesgo (8) y los determinantes sociales de la salud (DSS) (18).

Se emplearon métodos cuantitativos y cualitativos; las unidades de análisis se seleccionaron de manera intencional, según criterios de inclusión y exclusión preestablecidos.

***Criterios de inclusión:*** todos los productos comunicacionales y el contenido de sus mensajes relacionados con la prevención de la COVID-19 y el contexto de la pandemia destinados a la población general. Se consideraron los materiales alojados en los sitios web de los ministerios de salud de los 47 países y territorios (incluidos los departamentos de ultramar) de ALC (19), publicados desde el comienzo de la pandemia en cada país y que se encontraban disponibles y accesibles hasta el 28 de febrero del 2021. Se seleccionaron todos los productos presentes y accesibles en la web, sin distinción de formato y origen, en español, inglés, portugués, neerlandés y francés, según el idioma oficial de cada país.

En el caso de las redes sociales (Facebook y YouTube) y otros sitios oficiales de gobierno, solo se incluyeron los productos que tuvieran acceso directo desde las páginas de los ministerios de salud.

Cuando se constató un número muy reducido o nulo de productos comunicacionales en el sitio de Internet de la entidad gubernamental encargada de la salud, se incluyeron los productos contenidos en páginas gubernamentales específicas sobre la COVID-19 y otras páginas de gobierno que tuvieran una sección para la COVID-19; en caso de no contar con productos comunicacionales en esas fuentes, se recurrió a la página oficial en Facebook de la autoridad sanitaria del país o territorio en cuestión.

***Criterios de exclusión:*** productos comunicacionales y mensajes sobre prevención en el marco de la pandemia que estuvieran dirigidos exclusivamente a grupos específicos (niños, niñas, adolescentes, personas mayores, embarazadas, lactantes, personas de riesgo y con enfermedades preexistentes —crónicas o no—, pacientes en aislamiento, personas con síntomas y diagnóstico de COVID-19, deportistas o propietarios de animales domésticos, entre otros). También se excluyeron los productos destinados a centros escolares, y personal e instituciones de salud; documentos meramente normativos, epidemiológicos y sobre cobertura de vacunación; notas y comunicados de prensa, noticias, avisos y propaganda oficial; lineamientos, guías y orientaciones para la atención de pacientes y las pruebas de COVID-19; protocolos de bioseguridad específicos y recomendaciones destinados a instituciones educativas, comercios, restaurantes, gimnasios, eventos especiales y turísticos, transporte, bancos y empleos; blogs o mensajes de usuarios. Por último, se excluyeron los productos en lenguas indígenas, dialectos locales y otros idiomas no oficiales de ALC que no contaran con traducción a los idiomas oficiales incluidos en este estudio.

### Recolección de datos

La recogida de datos se llevó a cabo mediante una búsqueda sistemática realizada durante el mes de febrero del 2021. Para mejorar la confiabilidad y la reproducibilidad del estudio se utilizaron las siguientes palabras clave en el motor de búsqueda de Google: COVID-19, nombre del ministerio en el idioma oficial del país y nombre del país. También se realizó una búsqueda manual.

Se empleó un instrumento de recolección de información en línea basado en dimensiones y categorías preestablecidas, con un campo abierto para la información emergente. Se descargaron y guardaron todos los productos comunicacionales de los sitios web explorados.

Para mejorar la validez interna y mitigar posibles sesgos, se revisó al menos dos veces toda la información recogida, se eliminaron productos duplicados y se llevó a cabo una triangulación por investigador (20). De esta manera, también se revisaron discrepancias y se corrigieron posibles errores en la entrada de datos.

### Variables, dimensiones y categorías

Para el análisis cuantitativo se utilizaron las siguientes variables y categorías:

- Países de ALC según la subregión (19), con 3 categorías: América del Sur, América Central y México, y el Caribe.
- Tipo de producto (21), con 10 categorías: video (video de más de 30 segundos), *spot* (video de hasta 30 segundos), infografía, texto suelto, imagen con o sin texto, boletín, guía, audio, y material interactivo (enlaces a WhatsApp, teléfonos, chats en línea, buzón para sugerencias en línea, aplicaciones telefónicas o *apps*, y enlaces a teleconsultas).
- Tipo de soporte digital, con 4 categorías: sitio web del ministerio de salud (incluidos los enlaces a otras páginas de gobierno), del gobierno y específico sobre COVID-19 no perteneciente al área de salud, y página oficial de la autoridad sanitaria en Facebook.
- Consejos para la prevención de la COVID-19 según las orientaciones de la Organización Mundial de la Salud (OMS) para el público general (17). Se definió como “presente” cuando en al menos un producto comunicacional se hacía mención, o se incorporaba —de manera total o parcial— alguna de las siguientes 15 categorías: lavado de las manos con agua y jabón; lavado de las manos con gel hidroalcohólico; uso de mascarilla casera; uso de mascarilla quirúrgica o médica; distanciamiento social de al menos un 1 metro; distanciamiento social de más de 1 metro; evitar espacios cerrados o congestionados; reuniones al aire libre; ventilación de espacios cerrados; forma sana de toser o estornudar: no tocarse la cara, nariz u ojos; limpieza y desinfección de superficies y objetos; conocer los síntomas; cómo hacer el autoaislamiento; y cómo y dónde buscar atención médica.

Las dimensiones empleadas en el análisis cualitativo fueron:

- DSS intermedios propuestos por la OMS (18). Se definió como “presente” cuando en al menos un producto comunicacional se hacía mención o se incorporaba —de manera total, parcial o trasversal— alguna de las siguientes 4 categorías y sus respectivas subcategorías: factores psicosociales (salud mental) —incluidos elementos de comunicación de riesgo a partir del análisis de la infodemia (8, 22)—, circunstancias materiales (entorno), sistema de salud (vacunación contra la COVID-19), y factores biológicos y de comportamiento (hábitos saludables).
- DSS estructurales propuestos por la OMS (18), abordados desde un enfoque de diversidad (23, 24). Se definió como “presente” cuando en al menos un producto comunicacional se hacía mención o se incorporaba —de manera total, parcial o trasversal— alguna de las siguientes 4 categorías y sus respectivas subcategorías: inclusión de indígenas, afrodescendientes y migrantes; adaptación a diferentes lenguas, idiomas o dialectos; inclusión de personas con discapacidad; y enfoque de género.

### Análisis de los datos

Para analizar las variables cuantitativas se utilizaron frecuencias absolutas y relativas. Para los datos cualitativos se aplicó el análisis de contenido en dos etapas (25): análisis global para la familiarización con la información, y análisis en profundidad según las categorías y subcategorías preestablecidas y emergentes; se identificaron elementos visuales, audiovisuales, frases, palabras del título y del cuerpo del mensaje, y se generaron descripciones de acuerdo con la naturaleza del producto comunicacional.

Todos los elementos identificados se clasificaron y codificaron mediante la técnica manual de codificación de la información con la ayuda del programa informático ATLAS.ti (versión 6.2).

Se seleccionaron ejemplos de productos y sus respectivos enlaces para ilustrar el contenido de las categorías (cuadros A1, A2 y A3 del material suplementario).

Este estudio se basó en productos comunicacionales publicados en sitios en Internet y redes sociales de entidades gubernamentales con acceso libre y abierto, y no se trabajó con seres humanos, por lo que tanto el Comité Nacional de Bioética de la Investigación de Panamá como el Comité Institucional de Ética de las Investigaciones en Salud del Hospital Nacional de Clínicas de la Universidad Nacional de Córdoba de Argentina, dictaminaron su exención de evaluación.

## RESULTADOS

Después de aplicar los criterios de inclusión y exclusión, se seleccionaron para el análisis 3 253 productos comunicacionales, de ellos 1 825 (56,1%) se basaban en infografías, 532 (16,4%) en videos de más de 30 segundos, 228 (7,0%) en imágenes con texto, y el resto eran *spots* (146; 4,5%), textos sueltos (126; 3,9%), imágenes sin texto (16; 0,5%), boletines (42; 1,3%), guías (148; 4,5%), y materiales de audio (160; 4,9%) e interactivos (30; 0,9%), que se encontraron presentes en menos del 5% de los productos seleccionados.

Del total, 1 044 (32,1%) se encontraban en los sitios web de los ministerios o en sus enlaces a otros sitios gubernamentales, 1 078 (33,1%) en la red social Facebook oficial de los ministerios de salud, 598 (18,4%) en otros sitios de gobierno y 533 (16,4%) en sitios específicos de COVID-19 no pertenecientes al área de salud.

La mayor cantidad de productos informacionales se encontraron en sitios web de países de América Central y México (1 322; 40,7%), seguidos por los del Caribe (1 172; 36,0%) y América del Sur (759; 23,3%).

### La prevención de la COVID-19 en las orientaciones al público general

**FIGURA 1.**
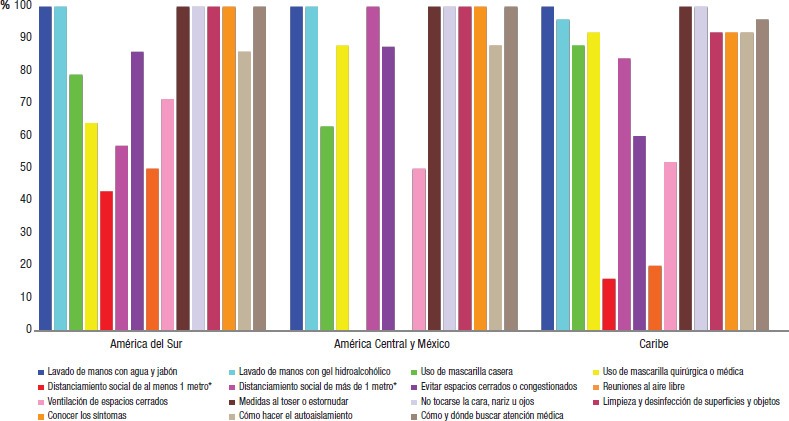
Proporción de países con presencia de al menos un producto comunicacional promovido por los gobiernos que se ajusta a las medidas preventivas de la COVID-19^a^, según la subregión de América Latina y el Caribe, 2021^b^

En todos los países y territorios de ALC se encontró al menos un producto comunicacional que incluyó algunas de las recomendaciones establecidas por la OMS (figura 1). La recomendación del lavado de manos con agua y jabón estuvo presente en al menos un producto en todos los países y territorios analizados.

La proporción de países que dieron la recomendación de mantener una distancia de más de 1 metro de otras personas en al menos un producto fue de 100% en América Central y México, de 84,0% en el Caribe; y de 57,1% en América del Sur; el resto de los países recomendaron el distanciamiento de al menos 1 metro.

Fue menor la proporción de países que abordaron en al menos un producto aspectos relacionados con el entorno, como las reuniones al aire libre: ninguno de América Central y México, el 20% en el Caribe y el 50% en América del Sur (figura 1) (cuadro A1, del material suplementario).

### Determinantes intermedios

En 46 países o territorios se abordó alguna de las categorías y subcategorías relacionadas con los DSS intermedios en al menos un producto comunicacional; dentro de la categoría de salud mental, la prevención de suicidio estuvo presente en al menos un producto de 4 países y dentro de la categoría de hábitos saludables, la prevención de enfermedades no transmisibles se encontró en materiales de 10 países (cuadro 1) (cuadro A2 del material suplementario).

Cabe destacar que en 35 (74,5%) de los países estudiados hubo al menos un producto con información errónea y en 13 (27,7%) no se encontró al menos un producto que abordara total o parcialmente la prevención de la infodemia (cuadro 1).

***Fuente:*** elaboración propia.

^a^ Según las orientaciones de la Organización Mundial de la Salud para la prevención de la COVID-19 destinadas al público general (17).

^b^ Se definió como presencia cuando en al menos un producto comunicacional se hacía mención, o se incorporaba —de manera total, parcial o transversal— alguna de las medidas preventivas de la COVID-19.

^*^ En el caso de los países en los que se encontraron productos comunicacionales con recomendaciones de diferentes medidas de distanciamiento, ya fueran de al menos 1 metro (incluido cuando no se especificó la distancia) o de más de 1 metro, se consideró la medida que tuvo mayor presencia.

### Determinantes estructurales

Como se muestra en el cuadro 2, a partir del análisis de los productos comunicacionales que aplicaban o incluían —de manera total, parcial o transversal— un enfoque de diversidad, se encontró que solo en 7 países se adaptó al menos un producto a lenguas indígenas, en 4 países se incluyó a personas indígenas en los mensajes y en 9 a personas con discapacidad; solo en 1 país se desarrollaron mensajes destinados a personas migrantes. Se observó que solo en 4 países se incluyó en al menos un producto alguna de las subcategorías definidas dentro del enfoque de género (cuadro A3 del material suplementario).

## DISCUSIÓN

Si bien se encontraron productos comunicacionales que dentro de sus temáticas abordaron las recomendaciones de la OMS para la prevención de la COVID-19, se observó un limitado enfoque desde los DSS, en especial en lo relacionado con la diversidad y el abordaje integral de la salud. Es preocupante que se hayan encontrado productos con errores, así como la escasa presencia de productos y mensajes dirigidos a prevenir la infodemia.

Un estudio previo mostró que en la crisis sanitaria provocada por el virus del Ébola, el nivel de comunicación fue mínimo, con un uso reducido de materiales en formato de multimedia (26). En contraste, en el presente estudio se constató que los recursos utilizados se basaron en diferentes formatos digitales con predominio de infografías y videos de más de 30 segundos, algo similar a lo encontrado por Villegas-Tripiana y colaboradores en un estudio que analizó las campañas de comunicación sobre la COVID-19 en España (27).

En cuanto a las medidas preventivas de la COVID-19 abordadas, se encontró una fuerte presencia de productos y mensajes sobre el distanciamiento social, el lavado de las manos, el uso de mascarillas y la salud respiratoria, consistente con lo encontrado en el estudio español antes citado (27). Otra similitud con ese estudio es que, si bien se encontraron algunos productos con recomendaciones sobre salud mental, hábitos saludables y otras prácticas de prevención de enfermedades no transmisibles, estos tuvieron un alcance limitado. Como plantean Mheidly y Fares (28), el cierre de clínicas y la demora de los turnos pueden empeorar las condiciones crónicas de salud, por lo que en este contexto, las redes sociales serían una herramienta estratégica para promover la salud y el bienestar.

En la mayoría de los países se brindó algún tipo de información relacionada con la vacunación contra la COVID-19, más de lo encontrado por Villegas-Tripiana y colaboradores, que abordaron la vacunación general (27); esto puede deberse a que ese estudio se llevó a cabo en una etapa temprana de la pandemia, cuando todavía no se avizoraban las vacunas. Esta discrepancia muestra cómo en el entorno dinámico de la pandemia, las medidas implementadas por los gobiernos son cambiantes y, por consiguiente, se deben modificar los mensajes comunicacionales difundidos.

**CUADRO 1.** Caracterización de los productos comunicacionales promovidos por los gobiernos, relacionados con determinantes sociales intermedios^a^ destinados al público general, por país o territorio de América Latina y el Caribe, 2021^b^

Factores analizados	Anguila	Antigua y Barbuda	Argentina	Aruba, Bonaire	Bahamas	Barbados	Belice	Bermudas	Bolivia	Brasil	Chile	Colombia	Costa Rica	Cuba	Curazao	Dominica	Ecuador	El Salvador	Granada	Guadalupe	Guatemala	Guyana	Guayana Francesa	Haití	Honduras	Islas Caimán	Islas Malvinas	Islas Turcas y Caicos	Islas Vírgenes	Islas Vírgenes Británicas	Jamaica	Martinica	México	Montserrat	Nicaragua	Panamá	Paraguay	Perú	Puerto Rico	República Dominicana	Saint Kitts y Nevis	San Vicente y las Granadinas	Santa Lucía	Suriname	Trinidad y Tabago	Uruguay	Venezuela
Factores psicosociales: salud mental																																													
Prevención de la infodemia																																															
Información errónea^c^																																															
Líneas de apoyo																																															
Salud mental y bienestar																																															
Estrés, miedo, pánico																																															
Depresión, ansiedad, duelo																																															
Pasatiempos																																															
Prevención de suicidio																																															
Contacto con seres queridos																																															
Estigma social, discriminación																																															
Prevención de violencia general																																															
Circunstancias materiales: entorno																																															
Al salir de casa																																															
Espacio público, recreación																																															
Compras en supermercados																																															
Transporte público																																															
Lugares cerrados y de trabajo																																															
Viajeros																																															
Higiene																																															
General y de alimentos																																															
Sistema de salud: vacunación contra la COVID-19																																															
Información general o específica																																															
Factores biológicos y de comportamiento: hábitos saludables																																															
Actividad física																																															
Dieta saludable																																															
Sueño adecuado																																															
Consumo de sustancias tóxicas																																															
Prevención de ENT^d^																																															

***Fuente:*** elaboración propia.

a Determinantes sociales de la salud intermedios según la definición de la Organización Mundial de la Salud (OMS) (18).

b El color naranja indica la presencia de al menos un producto comunicacional que menciona o incorpora de manera total, parcial o trasversal la categoría indicada. El color gris indica que no se detectó ningún producto comunicacional que mencione o incorpore de manera total, parcial o transversal la categoría indicada.

c Se incluyó la información errónea plasmada de manera explícita en por lo menos un producto comunicacional. Se consideró información errónea cualquier dato sobre el nuevo coronavirus y las medidas para la prevención de la COVID-19 que contradijeran lo definido y recomendado por la OMS o que se basaran en rumores (22) al momento de este estudio como: usar guantes; usar protectores faciales, oculares o mascarillas con válvulas; medicamentos para prevenir o tratar la COVID-19; practicar actividad física con mascarilla; usar mascarilla a veces o en ciertas ocasiones (solo cuando se presenta síntomas de enfermedad respiratoria o se realiza cuidados de personas enfermas); quitarse el calzado antes de entrar al hogar.

d ENT: enfermedades no trasmisibles.

**CUADRO 2.** Caracterización de los productos comunicacionales promovidos por los gobiernos, relacionados con determinantes sociales intermediosa destinados al público general, por país o territorio de América Latina y el Caribe, 2021^b^

Factores analizados	Anguila	Antigua y Barbuda	Argentina	Aruba, Bonaire	Bahamas	Barbados	Belice	Bermudas	Bolivia	Brasil	Chile	Colombia	Costa Rica	Cuba	Curazao	Dominica	Ecuador	El Salvador	Granada	Guadalupe	Guatemala	Guyana	Guayana Francesa	Haití	Honduras	Islas Caimán	Islas Malvinas	Islas Turcas y Caicos	Islas Vírgenes	Islas Vírgenes Británicas	Jamaica	Martinica	México	Montserrat	Nicaragua	Panamá	Paraguay	Perú	Puerto Rico	República Dominicana	Saint Kitts y Nevis	San Vicente y las Granadinas	Santa Lucía	Suriname	Trinidad y Tabago	Uruguay	Venezuela
Inclusión de personas indígenas, afrodescendientes y migrantes																																															
En productos generales																																															
Personas indígenas																																															
Personas afrodescendientes																																															
Productos específicos																																															
Para pueblos indígenas																																															
Para afrodescendientes																																															
Para migrantes																																															
Adaptación de productos a diferentes lenguas, idiomas o dialectos																																															
En lengua indígena																																															
En dos o más idiomas o dialectos																																															
Inclusión de personas con discapacidad (PcD) en los productos
Generales																																															
Específicos para PcD																																															
Generales en lenguaje de señas																																															
Enfoque de género																																															
Mujeres en protagónicos (icono de la campaña)																																															
Prevención de la violencia de género y doméstica																																															
Diversidad sexual																																															

***Fuente:*** elaboración propia.

a Determinantes sociales de la salud estructurales según la definición de la Organización Mundial de la Salud (18).

b El color naranja indica la presencia de al menos un producto comunicacional que menciona o incorpora de manera total, parcial o transversal la categoría indicada. El color gris indica que no se detectó ningún producto comunicacional que mencione o incorpore de manera total, parcial o transversal la categoría indicada.

Se encontraron importantes vacíos en cuanto a las temáticas y el tratamiento transversal de la diversidad en los productos comunicacionales destinados a la población general, con escasa adaptación a diferentes lenguas —en un contexto en el que gran parte de los países de ALC cuentan con poblaciones indígenas (29)—; esto confirma resultados similares encontrados en Taiwán (30).

Un hallazgo alarmante fue el escaso enfoque de género en los productos analizados, hecho ya descrito en parte en el estudio español (27). Estos factores —y otros abordados en este trabajo sobre el enfoque ajustado a la diversidad— desempeñan un papel clave en la aceptabilidad de la información y de las recomendaciones gubernamentales (31). Según otros autores (28) y las experiencias documentadas a partir de la epidemia de ébola (32), se requiere adaptar las intervenciones y los mensajes a los diferentes grupos y comunidades.

Como plantean Clark-Ginsberg y Petrun Sayers (12), la comunicación durante las crisis rara vez se adecua a satisfacer las necesidades de las poblaciones vulnerables. Sin embargo, estos grupos experimentan resultados aun más negativos, entre otras causas, por ser también más vulnerables a los efectos negativos de la infodemia que ha rodeado a la COVID-19.

Tanto en el presente estudio como en una investigación realizada en África (11), se encontraron mensajes con información no sustentada en datos científicos, en gran medida basados en rumores. Preocupa que una parte considerable de estos mensajes se publicaron en el 2020 y no se habían actualizado al revisarlos para esta investigación, una situación agudizada por los pocos productos informacionales específicos —o de mensajes dentro de productos generales— destinados a sensibilizar a la población sobre la importancia de combatir la infodemia. Este resultado concuerda con los de otro estudio realizado en países de América del Sur en el que se constata la falta de estrategias para enfrentar la infodemia desde los ministerios de salud de los países estudiados (33). Esta realidad debe ser objeto prioritario de futuros estudios y acciones.

A partir de los resultados y el análisis realizado, se proponen las siguientes recomendaciones de comunicación destinadas a los gobiernos de la Región para aplicar durante la pandemia de COVID-19.

### Políticas y estrategias de comunicación

- Elaborar un plan de comunicación actualizado sobre la COVID-19, con un componente de monitoreo ajustado tanto a las diferentes etapas de la pandemia en cada país como al avance del conocimiento en el mundo.
- Desarrollar estrategias que incluyan productos comunicacionales diseñados desde un enfoque de los DSS (18) para contribuir a la mejor recepción, decodificación, aceptación y adopción de los mensajes y, a su vez, promover mejores prácticas de prevención y cuidados.
- Incluir en la política un componente de comunicación de riesgo dirigido a la prevención de la infodemia (8), de manera trasversal, en todas las etapas de la pandemia.

### Medios de difusión

- Consolidar portales específicos para la COVID-19, cuyos mensajes, materiales y productos informacionales se mantengan actualizados y accesibles, y realizar un correcto ordenamiento de los mensajes según el público al que va dirigido, con una adecuada diferenciación de la información destinada al público general.
- Generar una estrategia unificada de publicación y amplificación entre los sitio web oficiales de salud y las redes sociales, a fin de lograr un mayor y mejor alcance de la información; este aspecto es importante, ya que resulta llamativo que en 10 de los países y territorios analizados no se halló información en los sitios web gubernamentales y se tuvo que recurrir a la red social Facebook para encontrarla.

### Contenido de los productos comunicacionales

- Revisar y ajustar los productos existentes y sus contenidos —en lugar de crear nuevos—, algo que podría mejorarse en los 47 países y territorios estudiados.
- Analizar los productos disponibles para evaluar si están diseñados desde un enfoque de comunicación de riesgo y de los DSS (18) y adaptados a las particularidades de los diferentes grupos de la población.
- Difundir mensajes mediante productos orientados a las necesidades de las personas y evitar que contengan información falsa o errónea.
- Elaborar productos generales aptos para todos los grupos poblacionales; si bien en este estudio no se analizó el efecto positivo que podría generar la alfabetización en salud (9) para la correcta recepción de los mensajes por parte de los destinatarios, esto deberá tomarse en cuenta dada la cantidad de productos encontrados basados en videos largos (de más de 30 segundos) e infografías, en comparación con otros recursos más sencillos como *spots* e imágenes, que son más fáciles de comprender por una mayor parte de la población.

Al analizar estas recomendaciones, se deben tomar en cuenta algunas limitaciones de este estudio, en particular su alcance concreto y que no se tomó en cuenta el posible paralelismo diferencial entre el contenido de los productos comunicacionales y el contexto epidemiológico, las diferentes etapas de avance de la pandemia y las medidas tomadas por los gobiernos en cada país.

Ciertos productos y mensajes de comunicación creados y difundidos por los ministerios de salud pudieron no llegar a registrarse o no estar disponibles en línea al momento de este estudio. Además, en este trabajo no se analizaron los productos difundidos por otras vías de comunicación, como la prensa escrita, la radio, la televisión y varias redes sociales. Para evitar o reducir este posible sesgo, en los casos de mínima o ninguna presencia de los mensajes buscados en las páginas web gubernamentales, se recurrió a la red social de Facebook de las entidades oficiales de salud.

Por otra parte, no se indagó qué tipo de productos de comunicación fueron efectivamente asimilados por parte de las personas a las que iban dirigidos, por lo que se requieren estudios que profundicen en la recepción de la información, en consonancia con la alfabetización en salud (9).

A pesar de esas limitaciones se logró caracterizar de manera global el contenido de los productos y mensajes comunicacionales dirigidos a la prevención de la COVID-19 impulsados por los gobiernos de ALC, a partir de una búsqueda suficientemente exhaustiva y representativa de todos sus países y territorios, un tema con muy pocos antecedentes.

Se puede concluir que, si bien se encontró una cantidad considerable de productos comunicacionales para la prevención de la COVID-19, estos no reflejaban un adecuado enfoque de diversidad y salud integral, además de detectarse en los mismos vacíos y errores.

Se requiere con urgencia que las autoridades gubernamentales y sanitarias de ALC revisen sus políticas de comunicación sobre la COVID-19 a fin de lograr un mayor y mejor alcance de sus mensajes a todos los sectores de la población. Se deben recoger datos sistemática y longitudinalmente para capturar las variaciones en el comportamiento de la pandemia y ajustar los mensajes consecuentemente.

Estas recomendaciones pueden servir, debidamente ajustadas, para la formulación de políticas y estrategias de comunicación en futuras pandemias y crisis sanitarias en la Región.

## Declaración.

Las opiniones expresadas en este artículo son responsabilidad de las autoras y no necesariamente reflejan las opiniones, políticas o posiciones oficiales de las instituciones a las cuales están afiliadas, ni los criterios ni la política de la *Revista Panamericana de Salud Pública / Pan American Journal of Public Health* y/o de la Organización Panamericana de la Salud.
